# How Simmons Became a “Regular”

**Published:** 1910-06

**Authors:** 


### How Simmons Became a "Regular."

(Lydston in the *Medical Sentinel*, May, 1910.)

CHICAGO, December 13, 1909.

Dr. Margaret Simmons appearing before me, a regularly commissioned notary public, for the county of Cook, State of Illinois, and being duly sworn, states as follows:

I was associated in practice with Dr. George H. Simmons from September, 1891, to April, 1892. I took charge of his practice whenever he was absent from Lincoln, Nebraska. In October or November, 1891, I matriculated for him for the winter term of lectures at Rush Medical College, Chicago, Illinois. He did not attend the lectures at said college and was absent from Lincoln, Nebraska, not longer than ten or twelve days in the fall of 1891, and only about ten or twelve days in the spring of 1892. In explanation of his ability to keep his college record clear he stated to me that he engaged a proxy to answer the quizzes and roll call.

(Signed) DR. MARGARET E. SIMMONS.

Subscribed and sworn to before me this 13th day of December, 1909.

(Signed) JOHN B. CHAPMAN,
(Seal.) Notary Public.

[Dr. Margaret E. Simmons, I understand, is Dr. Geo. H. Simmons' divorced wife.—D.]

---

A SIMPLE AND EFFICIENT OPERATION FOR HEMORRHOIDS.—N. Porritt thus describes an operation recently performed by him. The sphincter having been stretched, one of the hemorrhoidal masses was seized with vulsellum forceps, pulled well out of the anus and encircled with a loop of loose purse-string suture of Pagenstecher's thread. Above, the suture entered the healthy mucosa beyond the hemorrhoids; below, it took up that just within the anus while at each side it was inserted far enough apart to allow the blades of the crushing instrument to grasp the base or pedicle of the pile seized. For crushing he used an appendicitis clamp, but a pair of hemostatic forceps with blades long enough to overlap the base of the pile would answer. When the clamp is released a broad band of thin tissue connects the pile with the rectum. This pedicle is now folded on itself by giving a half-turn to the pile, the clamp is reapplied, and the crushing is repeated. When the clamp has been removed something like a pedicle has been made, but another half-turn and another crushing leave only a fine pedicle of crushed tissue. The pile is then clipped away through this pedicle, which is tied with a fine ligature beyond the clamp. The stump of crushed tissue with the ligature upon it is then buried by drawing tight and tying the purse-string suture already inserted. Other portions of the mass were isolated and removed in the same way. The patient did well and got up in two weeks after operation. It is now five months after operation and there has been no after-contraction. In order to insert each separate purse string and get a good grip laterally with it a bridge of tissue must necessarily be left between each group of pile dealt with. The little bridge is a safeguard against destroying too much of the involved structures and while not seriously militating against the efficiency of the operation it certainly makes the occurrence of after-contraction improbable.—*Lancet, in N. Y. Med. Record.*

